# Should preventive antibiotics be used in patients with acute stroke? A systematic review and meta-analysis of randomized controlled trials

**DOI:** 10.1371/journal.pone.0186607

**Published:** 2017-10-19

**Authors:** Feng Zheng, Niklas von Spreckelsen, Xintong Zhang, Pantelis Stavrinou, Marco Timmer, Christian Dohmen, Roland Goldbrunner, Fang Cao, Qiang Zhang, Qishan Ran, Gang Li, Ruiming Fan, Shengtao Yao, Boris Krischek

**Affiliations:** 1 Department of Neurosurgery, Affiliated Hospital of Guangdong Medical University, Zhanjiang, Guangdong, China; 2 Department of Neurosurgery, University Hospital of Cologne, Cologne, Germany; 3 Department of Neurosurgery, The Second Clinical Medical School of Inner Mongolia University for the Nationalities (Inner Mongolia Forestry General Hospital), Inner Mongolia, China; 4 Department of Neurology, University Hospital of Cologne, Cologne, Germany; 5 Department of Cerebrovascular Disease, the first affiliated hospital of Zunyi Medical College, Zunyi, Guizhou, China; Azienda Ospedaliera Universitaria di Perugia, ITALY

## Abstract

**Background:**

Infection is a common complication in acute stroke. Whether or not preventive antibiotics reduce the risk of infection or even lead to a favorable outcome and reduction of mortality after a stroke still remains equivocal. This review was performed to update the current knowledge on the effect and possible benefits of prophylactic antibiotic therapy in patients with stroke.

**Methods:**

A systematic review and meta-analysis of preventive antibiotics`effect on the incidence of infection, favorable outcome (mRS≤2) and mortality in patients with acute stroke is performed with relevant randomized controlled trials.

**Results:**

Six studies were identified, involving 4125 participants. Compared with the control group, the treated groups were significantly less prone to suffer from early overall infections [RR = 0.52, 95%CI (0.39, 0.70), p<0.0001], early pneumonia [RR = 0.64, 95%CI (0.42, 0.96), p = 0.03] and early urinary tract infections [RR = 0.35, 95%CI (0.25, 0.48), p<0.00001]. However, there was no significant difference in overall mortality [RR = 1.07, 95%CI (0.90, 1.27), p = 0.44], early mortality [RR = 0.99, 95%CI (0.78, 1.26), p = 0.92], late mortality [RR = 1.12, 95%CI (0.94, 1.35), p = 0.21] or favorable outcome [RR = 1.00, 95%CI (0.92, 1.08), p = 0.98].

**Conclusion:**

Although preventive antibiotic treatment did reduce the occurrence of early overall infections, early pneumonia and early urinary tract infection in patients with acute stroke, this advantage was not eventually translated to a favorable outcome and reduction in mortality. Future studies are warranted to identify any subgroup of stroke patients who might benefit from preventive antibiotic treatment.

## Introduction

Infection is a common complication in the acute phase after a stroke and diagnosed in 21% to 65% of patients, with the most common infections being pneumonia and urinary tract infections [1–7]. Many studies have shown infections in patients with a stroke to be associated with poor short- and long-term functional outcome as well as a higher mortality [4, 8–10]. While this view is well established, a few studies failed to support this hypothesis [11, 12].

Several randomized controlled trials (RCTs) have assessed the preventive use of antibiotics in patients with acute stroke with conflicting results [11, 13–15]. A meta-analysis with pooled data from 506 stroke patients subsequently showed a 14% reduction in all infections with preventive antibiotics, but their effectiveness in decreasing post-stroke pneumonia, disability, and mortality was equivocal [16]. Whether or not preventive antibiotics reduce the risk of poor functional outcome after stroke still remains uncertain [16]. Due to the lack of sufficient evidence to prove it effective, existing guidelines do not lend support to the use of prophylactic antibiotics in patients who have had a stroke [17].

Recently, a systematic review [18] following the publications of two large RCTs updates the knowledge regarding the effect of prophylactic antibiotics treatment on post-stroke infections and the occurrence of mortality among adult acute stroke patients. Nevertheless, the effect of preventive antibiotics on early infections, early and late mortality, outcome in ischemic and hemorrhagic stroke, as well as the length of hospital stay still remain uncertain. [18–20]. Therefore, we conduct a systematic review and meta-analysis with randomized controlled trials to better understand the effect of preventive antibiotics in patients with acute stroke.

## Methods

### Search criteria

This systematic review and meta-analysis was conducted in accordance with PRISMA guidelines [21]. All full text RCT, analyzing the incidence of infections, mortality, and functional outcome of preventive antibiotic therapy versus control (placebo or open control) in published studies were included. Case reports, non-randomized studies, comments, letters, editorials, protocols, guidelines, and animal studies were excluded. The Pubmed, Embase, and Cochrane library were searched for English-language articles published prior to Feb, 27, 2017. Unpublished studies were excluded. The data bases were searched using appropriate keywords and MeSH terms (See S3 File). Titles, abstracts, and subject headings were searched. The reference lists of all included articles and review papers were scrutinized for additional publications.

### Outcome

The primary outcome was any early infection, early pneumonia, early urinary tract infection (UTI), secondary outcome included late infection, overall mortality, early mortality, late mortality, the length of hospital stay, favorable outcome defined by the modified Rankin Scale (mRS) at the end of follow-up in patients with acute stroke, acute ischemic stroke and acute hemorrhagic stroke. Early infection was defined as occurrence of infection within the first 14 days after onset of symptoms. Early mortality was defined as occurrence of mortality within the first 14 days after onset of symptoms. Late infection was defined as occurrence of infection between early and the end of follow-up after onset of symptoms. Late mortality was defined as occurrence of mortality after 14 days or more after onset of symptoms. Overall mortality was defined as early mortality plus late mortality. Favorable outcome was defined as the mRS ≤2 at the end of follow-up.

### Data collection and extraction

Two reviewers (F.Z. and X.Z.) independently screened the titles of all papers identified in the search to eliminate duplicate references, reviewed abstracts and selected RCTs according to the inclusion criteria.

The following information was extracted from the included studies: (1) general information: author names and publication year; (2) baseline information: study design, inclusion and exclusion criteria, patients’ characteristics, intervention characteristics, the definition of infection; (3) the data of infection: the number of early and late infection events at the end of follow-up, as well as the number of pulmonary- and urinary tract infections in patients with early infection; (4) the data of mortality: the number of early and late mortality events at the end of follow up (5) the data of functional outcome: the number of favorable functional outcomes at the end of follow-up. Due to different definitions of infection throughout the studies (See S1 File), we adopted the definition used by each investigator. If both data for intention-to-treat (ITT) and per protocol (PP) were available, the ITT data was used to avoid preselection bias and avoid data influenced by “drop outs” through early death in the PP-population. When algorithm-based diagnosis and physician-based diagnosis of post-stroke infections were both available, the algorithm-based diagnosis was used to minimize detection bias.

### Analysis of the quality of the studies

The quality of the eligible studies was assessed using the Cochrane Collaboration’s tool for assessing risk of bias. Sequence generation, allocation sequence concealment, blinding, incomplete outcome data, selective outcome reporting and other potential sources of bias were evaluated. In accordance with the above mentioned tool, the risk for bias was set as either low, high or unclear (indicating either lack of information or uncertainty over the potential for bias) [22].

### Statistical analysis

The data included in the present study is dichotomous, the relative risk (RR) and 95% confidence intervals (CI) were therefore calculated as the pooled mean effect size estimate using the Mantel–Haenszel method. The present meta-analysis was carried out using REVMAN software (version 5.3 for Windows. Copenhagen: The Nordic Cochrane Centre, the Cochrane Collaboration). Quality assessment of the included studies was done independently by two reviewers. Statistical heterogeneity was measured using c^2^ and I^2^ statistics. Due to the existence of heterogeneity in study design (double-blind versus open-label studies), type of antibiotic therapy (adequately covering all pathogens in post-stroke infections versus mostly chosen for neuroprotective properties) and definitions of infection, we chose a random-effects model for the pooled analyses. At the recommendation of the Cochrane Statistical Methods Group [23], a significance level of P value of heterogeneity was set at 0.1, and the I^2^ statistic was interpreted as follows: 0% to 40% as low heterogeneity; 30% to 60% as moderate heterogeneity; 50% to 90% as substantial heterogeneity; and 75% to 100% as considerable heterogeneity. Statistically significant heterogeneity was considered present at p<0.10 and I^2^>50%. In this case, sensitivity analysis, which excludes the study contributing most to the heterogeneity, was employed to identify the robustness of the result.

## Results

Following the literature search scheme and characteristics of the eligible references

**Fig 1:**
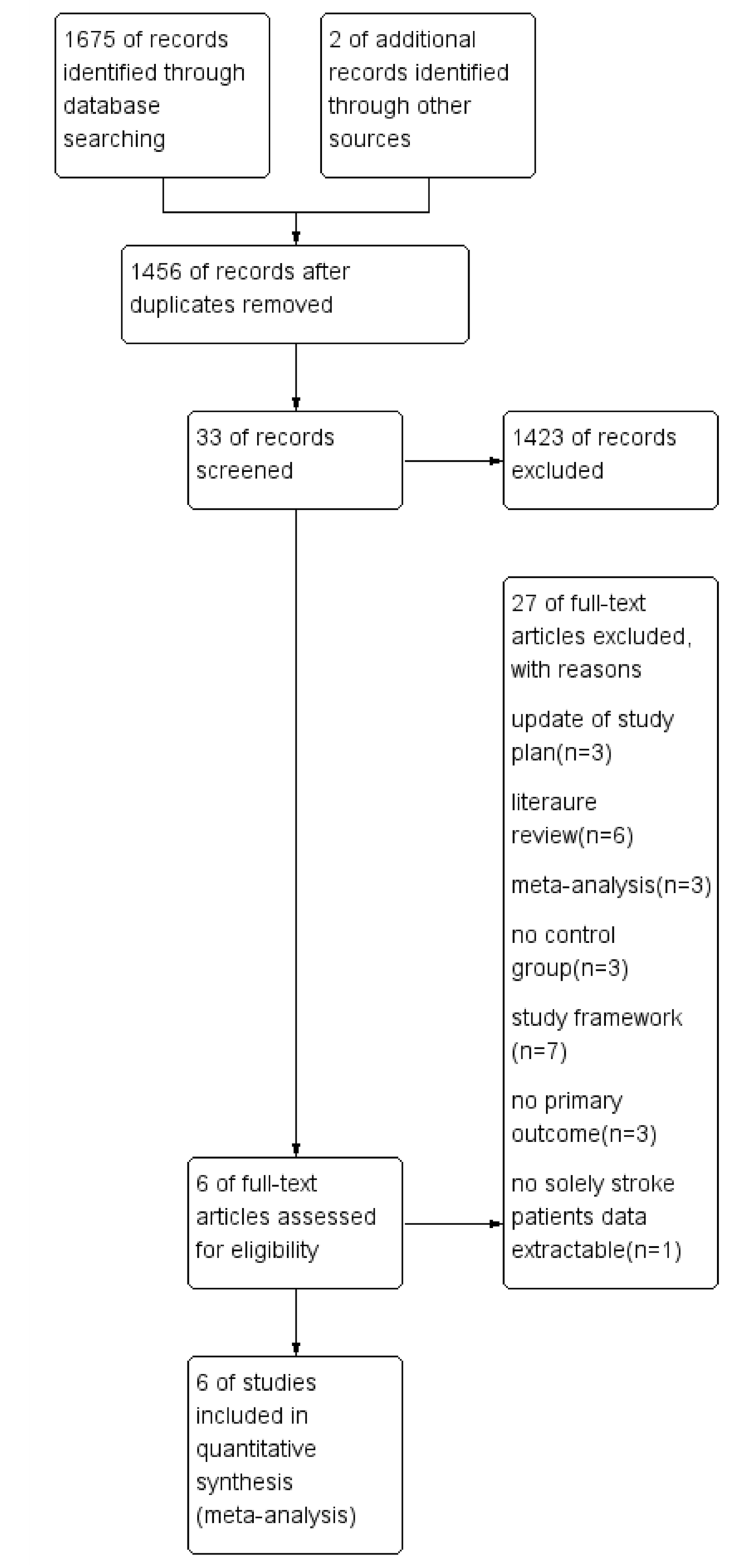
Literature search scheme.

As shown in Fig 1, we searched the Cochrane Library, PubMed database and EMBASE database and obtained an initial pool of 1677 references. Preliminary screening eliminated 221 duplicate references, and perusal of the titles and abstracts eliminated 1423 more references that did not meet the eligibility criteria. Examination of the full text eliminated 27 more references. These screening steps resulted in the identification of 6 studies involving a total of 4125 patients that were eligible for meta-analysis and systematic review [11, 13, 15, 19, 20, 24]. All patients included in these 6 studies were at least 18 years old. The characteristics of the 6 studies and the detailed outcome measurement are shown in Tables 1 and 2.

**Table 1:** The characteristics of the six included studies.

Study	Patient(n)		intervention	Method	Participant	Primary outcome	Secondary outcome	Analysis method	Diagnosis method
	P.A.	control							
**Chamorro 2005**[11]	67	69	Intravenous levofloxacin 500 mg/100 mL/d, for 3 days	randomized, double-blind	age>18 years, nonseptic ischemic or hemorrhagic stroke enrolled within 24 hours from clinical onset	the difference in early infection at first 7 days after stroke	neurological outcome and mortality at day 90	Not mentioned	algorithm-based
**Harms, 2008**[13]	39	40	Intravenous moxifloxacin 400 mg/d for 5 days	randomized, double-blind	age>17 years, ischaemic stroke in MCA territory and NIHSS ≥ 12 within 9 to 36 hours after onset	Infection rate within 11 days after stroke onset	neurological outcome, survival, development of stroke-induced immunodepression, and induction of bacterial resistance at day 180	ITT and PP	algorithm-based
**Kalra, 2015**[19]	615	602	amoxicillin or co-amoxiclav, together with clarithromycin for 7 days	cluster-randomised, open-label controlled trial with masked endpoint assessment	Page>18 years, confirmed diagnosis of new stroke (ischemic or hemorrhagic) with onset of symptoms within 48 hour at recruitment, and dysphagia	post-stroke pneumonia in the first 14 days	NIHSS score at 14 days, death at 14 and 90 days, functional outcome at 90 days defined by mRS, CDT-positive diarrhoea, MRSA colonisation, health-related; quality of life	ITT	Both algorithm-based and Physician-based available
**Kohler, 2013**[24]	47	48	Intravenous minocycline 100 mg/12 hour for a total of 5 doses	randomized open-label blinded end point evaluation	age> 18 years, onset of symptoms of stroke (ischemic or hemorrhagic) within 24 hours of administration of the trial intervention, any measurable neurological deficit on NIHSS, ability to provide informed consent.	survival free of handicap (mRS≤2) at day 90.	Categorical shift in mRS at day 90,mean NIHSS at day 7,mean Barthel Index at day 90, fever (temperature>38°C) at day 7	ITT and PP	Not mentioned
**Schwarz, 2008**[15]	30	30	Intravenous mezlocillin 2 g and sulbactam 1 g every 8 hours for 4 days	randomized, open-label	age≥18 years, ischemic stroke with onset of symptoms < 24 hours ago, bedridden (mRS > 3), an estimated premorbid mRS < 2 and stable deficits	incidence and height of fever at first 10 days	rate of infection at day 10 and clinical outcome at day 90	Not mentioned	algorithm-based
**Westendorp, 2014**[20]	1268	1270	intravenous ceftriaxone 2 g/d for 4 days	multicenter, randomized, open-label trial with masked endpoint assessment	age≥18 years, had clinical symptoms of a stroke(ischemic or hemorrhagic), onset of symptoms less than 24h ago, NIHSS score ≥1.	functional outcome at 3 months after stroke	death, infection rates, antimicrobial use at discharge, and length of hospital stay	ITT	Both algorithm-based and Physician-based available
**Total**	2066	2059							

CDT: Clostridium difficile toxin, ITT: intention-to-treat, mRS: modified Rankin Scale, MRSA: meticillin-resistant Staphylococcus aureus, NIHSS: National Institutes of Health Stroke Scale, P.A: preventive antibiotics, PP: per protocol

**Table 2:** The outcome of the six included studies.

study	Early infection (%)		Early pneumonia (%)		Early UTI (%)		Early mortality (%)		Late infection (%)		Late mortality (%)		Overall mortality (%)		Favorable function outcome (%)	
	P.A.	control	P.A.	control	P.A.	control	P.A.	control	P.A.	control	P.A.	control	P.A.	control	P.A.	control
**Chamorro, 2005**[11]	11	13							9	10	16	9	16	9		
	-16.40%	-18.80%	NA	NA	NA	NA	NA	NA	-13.40%	-14.50%	-23.90%	-13.00%	-23.90%	-13.00%	NA	NA
**Harms, 2008**[13]	6	13	3	8	3	5	3	4	6		3	3	6	7		
	-15.40%	-32.50%	-7.70%	-20.00%	-7.70%	-12.50%	-7.70%	-10.00%	-15.40%	NA	-7.70%	-7.50%	-15.40%	-17.50%	NA	NA
**Kalra, 2015**[19]	93	97	71	52	15	39	62	56	56	56	122	102	184	158	109	121
	-15.10%	-16.10%	-11.50%	-8.60%	-2.40%	-6.50%	-10.10%	-9.30%	-9.10%	-9.30%	-19.80%	-16.90%	-29.90%	-26.20%	-17.70%	-20.10%
**Kohler, 2013**[24]	1	9	1	2	0	7	1	1	2		1	1	2	2	29	33
	-2.10%	-18.80%	-2.10%	-4.20%	0%	-14.60%	-2.10%	-2.10%	-4.30%	NA	-2.10%	-2.10%	-4.30%	-4.20%	-61.70%	-68.80%
**Schwarz, 2008**[15]	15	27	7	10	8	18	1	3	2		1	3	2	6	0	0
	-50%	-90.00%	-23.30%	-33.30%	-26.70%	-14.60%	-3.30%	-10.00%	-6.70%	NA	-3.30%	-10.00%	-6.70%	-20.00%	0%	0%
**Westendorp, 2014**[20]	40	89	23	34	16	60	57	61	131		74	75	131	136	781	763
	-3.20%	-7.00%	-1.80%	-2.70%	-1.30%	-4.70%	-4.50%	-4.80%	-10.30%	NA	-5.80%	-5.90%	-10.30%	-10.70%	-61.60%	-60.10%
**Total**	166	248	105	106	42	129	124	125	206	66	217	193	341	318	919	917
	-8.00%	-12.00%	-5.10%	-5.10%	-2.10%	-6.50%	-6.00%	-6.10%	-10.00%	-9.80%	-10.50%	-9.40%	-16.50%	-15.40%	-44.50%	-44.50%

mRS: modified Rankin Scale, P.A.: preventive antibiotics, UTI: urinary tract infection, Favorable function outcome was defined as the mRS≤2 at the end of follow-up.

### Analysis of the quality of eligible references

**Fig 2:**
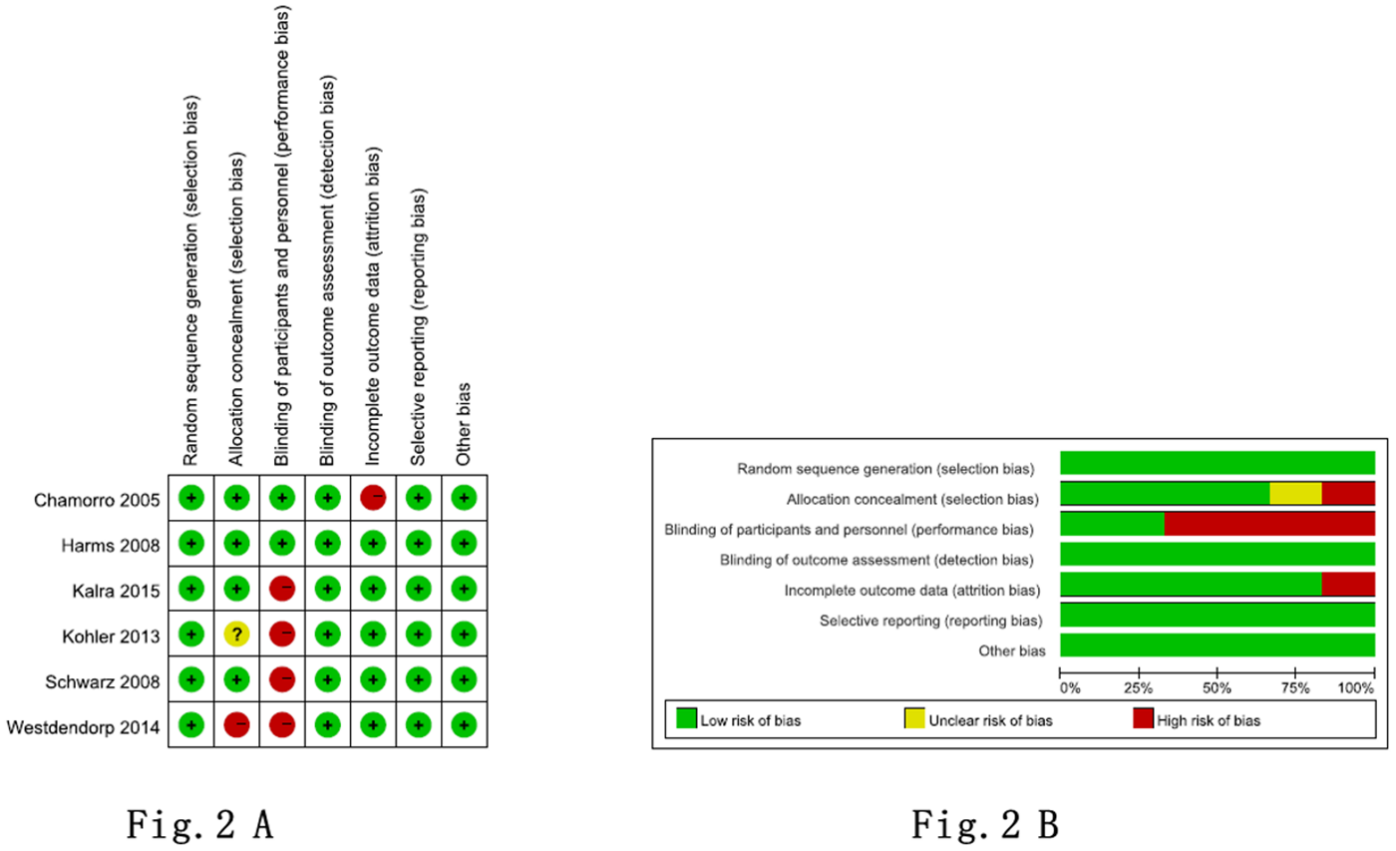
The quality analysis of all 6 studies. (A) Summary of the risk of bias in all included 6 studies. (B) Risk of bias graph: judgements about each risk of bias item presented as percentages across all included 6 studies.

We evaluated the eligibility criteria of the 6 identified studies using the Cochrane Collaboration tool. The quality analysis of all 6 studies is shown in Fig 2. Briefly, randomization methods were described in all 6 studies [11, 13, 15, 19, 20, 24] and allocation concealments were adequate in 4 studies [11, 13, 15, 19]. For blinding, 6 studies used blind observers to assess outcome [11, 13, 15, 19, 20, 24], while blinded for carers or patients were used in 2 studies [11, 13]. In addition, 5 studies reported the complete outcome [13, 15, 19, 20, 24]. Finally, none study had selective outcome reporting.

### Primary outcomes

All primary outcomes were significantly influenced by preventive antibiotics (Table 3, forest plots in S2 File).

**Table 3:** Summary of pooled data comparing preventive antibiotic group and control group in adult patients with acute stroke.

	Event (n)		Test for Heterogeneity		Test for Overall Effect		RR(95%CI)
Primary outcomes	Antibiotic	Control	I^2^	P	Z	P	
**Early infection**							
**Before S.A.**	**166(2066)**	**248(2059)**	**69%**	**0.007**	**2.63**	**0.008**	**0.60 (0.41, 0.88)**
**After S.A.**	**73 (1451)**	**151(1457)**	**21%**	**0.28**	**4.40**	**<0.0001**	**0.52 (0.39, 0.70)**
**Early pneumonia**							
**Before S.A.**	**105(1999)**	**106(1990)**	**51%**	**0.08**	**0.85**	**0.40**	**0.81 (0.51, 1.31)**
**After S.A.**	**34 (1384)**	**54 (1388)**	**0%**	**0.86**	**2.15**	**0.03**	**0.64 (0.42, 0.96)**
**Early urinary tract infection**	**42(1999)**	**129(1990)**	**0%**	**0.46**	**0.28**	**<0.00001**	**0.35 (0.25, 0.48)**
**Secondary outcomes**							
**Overall mortality**	**341(2066)**	**318(2059)**	**12%**	**0.34**	**0.76**	**0.44**	**1.07 (0.90, 1.27)**
**Early mortality**	**124(1999)**	**125(1990)**	**0%**	**0.84**	**0.10**	**0.92**	**0.99 (0.78, 1.26)**
**Late mortality**	**217(2066)**	**193(2059)**	**0%**	**0.61**	**1.26**	**0.21**	**1.12 (0.94, 1.35)**
**Favorable outcome**	**919(1960)**	**917(1950)**	**10%**	**0.33**	**0.02**	**0.98**	**1.00 (0.92, 1.08)**
**Outcome in ischemic stroke**	**690(1127)**	**674(1135)**	**0%**	**0.51**	**0.88**	**0.38**	**1.03 (0.96, 1.10)**
**Outcome in hemorrhagic stroke**	**63 (148)**	**61 (132)**	**0%**	**0.73**	**0.64**	**0.52**	**0.92 (0.71, 1.19)**

RR: the relative risk, CI: confidence interval, S.A.: sensitivity analysis

#### Early infection

In the six studies analyzing the risk to suffer from any early infection proved to be significantly lower in the antibiotics group [RR = 0.60, 95% CI (0.41, 0.88), p = 0.008]. Due to the existence of significant heterogeneity (p = 0.007; I^2^ = 69%), a sensitivity analysis was employed to assess the robustness of the findings. After exclusion of one trial by Kalra et al [19], the overall significance still remains [RR = 0.52, 95%CI (0.39, 0.70), p < 0.0001], without substantial heterogeneity (P = 0.28, I^2^ = 21%).

#### Early pneumonia

In the five studies which analyzed early pneumonia [13, 15, 19, 20, 24] initially no significant difference between both groups was found. [RR = 0.81, 95%CI (0.51, 1.31), p = 0.40]. However, in this subgroup the heterogeneity proved to be high (p = 0.08, I^2^ = 51%). After excluding the study performed by Kalra et al. (2015) which only included patients with dysphagia and contributed most to the heterogeneity, the heterogeneity lowered to (p = 0.86; I^2^ = 0%) and a significantly better outcome in the antibiotic group regarding early pneumonia was shown. [RR = 0.64, 95% CI (0.42, 0.96), p = 0.03].

#### Early urinary tract infection

Five studies recorded the event of early urinary tract infection (UTI) [13, 15, 19, 20, 24]. A significant advantage for the group treated with preventive antibiotics was shown. [RR = 0.35, 95% CI (0.25, 0.48), p < 0.00001] without substantial heterogeneity (p = 0.46, I^2^ = 0%).

### Secondary outcomes

None of the defined secondary outcomes were positively influenced by the preventive use of antibiotics. The meta-analysis showed no significant difference in overall mortality, early mortality, late mortality, the length of hospital stay or favorable outcome. (Table 3, forest plots in online supplements).

#### Overall mortality

The overall mortality at the end of follow-up was reported in all six studies [11, 13, 15, 19, 20, 24]. The number of overall mortality at the end of follow-up was 341 out of 2066 (17%) in the preventive antibiotics group versus 318 out of 2059 (15%) in the control group, with no significant difference [RR = 1.07, 95% CI (0.90, 1.27), p = 0.44], and no substantial heterogeneity. (p = 0.34; I^2^ = 12%).

#### Early mortality

Five studies recorded the event of early mortality in patients with acute stoke [13, 15, 19, 20, 24]. Both in the antibiotic group and the control group the early mortality rate was 6% (124 of 1999 vs. 125 of 1990) and no significant difference or heterogeneity was found. [RR = 0.99, 95% CI (0.78, 1.26), p = 0.92] (p = 0.84, I^2^ = 0%).

#### Late mortality

All six studies recorded the event of late mortality [11, 13, 15, 19, 20, 24]. In the preventive antibiotics group the number of late mortality events was 217 out of 2066 (11%) versus 193 out of 2059 (9%) in the control group. No significant difference regarding late mortality or substantial heterogeneity was found. [RR = 1.12, 95% CI (0.94, 1.35), p = 0.21] (p = 0.61, I^2^ = 0%).

#### The length of hospital stay

Two studies analyzed the correlation between the length of hospital stay and the administration of prophylactic antibiotic treatment, which present the comparison of days in hospital between antibiotic group and control group as 26 (12–55) vs 19 (9–43) and 6 (3–10) vs 6 (3–11) [19, 20]. No significant difference in the length of hospital stay was found between the groups in either of the two studies, although further quantitative analysis was not possible in the present study due to unavailable detailed data.

#### Favorable outcome

Favorable outcome was defined as mRS ≤ 2 at the end of follow-up and was assessed in four studies [15, 19, 20, 24]. In both groups 47% of the patients had a favorable outcome. (919 of 1960 antibiotics vs. 917 of 1950 control) and no significant difference in outcome was found. [RR = 1.00, 95% CI (0.92, 1.08), p = 0.98], without substantial heterogeneity (p = 0.33, I^2^ = 10%).

#### Difference in outcome in hemorrhagic vs. ischemic stroke

Three studies [15, 20, 24] analyzed the outcome for ischemic stroke and could not show a significant difference in favorable outcome (61%, 690 / 1127 antibiotic group vs. 59%, 674 / 1135 control group). [RR = 1.03, 95% CI (0.96, 1.10), p = 0.38], without substantial heterogeneity (p = 0.51, I^2^ = 0%).

Two studies [20, 24] reported hemorrhagic strokes and failed to show significant effect of antibiotics on favorable outcome as well. (63 out of 148 (41%) antibiotic group versus 61 out of 132 (46%), [RR = 0.92, 95% CI (0.71, 1.19), p = 0.52], without substantial heterogeneity (p = 0.73, I^2^ = 0%).

#### Publication bias

The publication bias was presented by funnel plots and examined by Eggers’ tests. No significant publication bias was found as a whole (Table 4).

**Table 4:** Publication bias assessment of this meta-analysis.

Outcome	Egger`s test (t-value)	P-value
**Early infection**		
**Before S.A.**	-1.28	0.269
**After S.A.**	-0.48	0.663
**Early pneumonia**		
**Before S.A.**	-2.00	0.139
**After S.A.**	-1.24	0.340
**Early urinary tract infection**	-0.27	0.802
**Overall mortality**	-0.44	0.684
**Early mortality**	-1.44	0.247
**Late mortality**	-0.29	0.786
**Favorable outcome**	-4.36	0.143
**Outcome in ischemic stroke**	NA	NA
**Outcome in hemorrhagic stroke**	NA	NA

S.A.: sensitivity analysis

## Discussion

Infections are the most common medical complication occurring in patients with stroke and are considered to be a key cause of mortality [25]. Pneumonia occurs in 11%-22%, and the risk of suffering from a UTI is even higher (16–24%) [6, 26]. The etiology of post-stroke pneumonia appears to be multifactorial. Invasive maneuvers, such as bronchoscopic diagnostics, aspiration of nasopharyngeal secretions caused by a decreased level of consciousness and reduced bulbar reflexes with oropharyngeal dysphagia [27–29], or feeding tube placement [12], could all contribute to a higher risk of pneumonia. In addition, old age, urinary incontinence, bedridden state with crural weakness, history of chronic obstructive pulmonary disease, diabetes, and stroke severity have been identified to be independent predictors of post-stroke nosocomial infection [30, 31].

In the current study, the difference in the outcome of early pneumonia between the two groups was initially not significant, but with substantial heterogeneity. To reduce substantial heterogeneity and also make the result more robust, sensitivity analysis was employed in accordance with the Cochrane handbook for systematic reviews of interventions [23]. After the significant heterogeneity had disappeared after sensitivity analysis, a significantly better outcome in the antibiotic group could be proven [RR = 0.64, 95% CI (0.42, 0.96), p = 0.03], which is different from a previously published systematic review by Liu et al [18]. This may be due to the different inclusion criteria. Compared with Liu et al`s publication, two studies were not included in the present meta-analysis. One is a non-English-language study [32]. The other did not provide efficiency data to analyze the effects of preventive antibiotics treatment on post-stroke infections and was therefore excluded [33]. Furthermore, we included one more eligible RCT study [24]. A special focus on this is warranted for future research.

Pneumonia is regarded as a predictor of poor outcome and mortality in patients after acute stroke. Several studies have reported that the occurrence of infections after stroke correlates with poor functional outcome and mortality [10, 31]. However, in the present meta-analysis, despite the significant reduction in early and late infection rate through preventive antibiotics, this advantage did not eventually translate to a more favorable outcome or a reduction of early or late mortality. This might support the findings of Vargas et al (2006) who showed that infections after stroke if treated promptly might not be an independent outcome factor, but merely an indicator of the stroke’s severity [12].

In previous experimental stroke studies, two antibiotics have been shown to be effective on functional outcome. One is minocycline, which seems to have several anti-inflammatory effects, reduces microglial activation, inhibits apoptotic cell death, and has a favorable effect on outcome in experimental stroke studies [34]. However, in a subsequent human research and meta-analysis, preventive intravenous minocycline did not affect the functional outcome in patients after acute stroke [24]. Another potential neuroprotective antibiotic is ceftriaxone, which has been reported reducing mortality and neurological deficits in a rat model of ischemic stroke [35]. In a large clinical trial with 2550 participants after acute stroke however this could not be confirmed for humans. Interestingly, in another randomized controlled trial, prophylactic administration of mezlocillin plus sulbactam was found to be likely associated with a better clinical outcome [15]. However, this result needs to be interpreted with caution because of a relatively small sample of 60 patients.

Correlation between the length of hospital stay and the administration of prophylactic antibiotic treatment had been described in two of the six included articles with a total of 3755 participants [19, 20]. Westendorp et al. (2015) clarified the length of hospital stay did not differ between two groups. Kalra et al. (2015) even found that patients in the antibiotics group had longer stays in hospital than control patients, although this difference was not significant. This indicates that the administration of prophylactic antibiotics may not shorten the hospital stay.

Several factors may bias the findings of the current study. Five studies used preventive antibiotic therapy that covered the common causative organisms in post-stroke infections [11, 13, 15, 19, 20]. However, minocycline, a kind of antibiotic with inadequate microbiological coverage, was used in order to investigate a possible neuroprotective effect in one study [24], which might have weakened the effect of preventive antibiotics on infection and even outcome. Different definitions for the diagnosis of infection were employed in all these six studies [11, 13, 15, 19, 20, 24], and less strict definitions might underestimate the number of infection events, especially in studies with an open-label design [36].

The mortality rates were very low in three included studies [15, 20, 24], ranging from 4% to 13%, which is less than previously reported mortality rates of between 15% to 25% in patients with acute stroke [37]. In one of these studies, patients with a life expectancy of less than 90 days were excluded [15], which obviously could skew reported results of mortality. The selection of patients with different extents of stroke is also a major variable in these studies.

The different effect of preventive antibiotic therapy in patients with a mild stroke versus a severe stroke is hard to grasp. Only two of these six included studies were double-blinded [11, 13], three of the other four studies used an open-label design [19, 20, 24], and in the remaining one study, although infection was assessed blindly, other outcomes were not [15]. Once a medical treatment differs between two groups, knowledge of the intervention in a trial might affect outcome. Although case fatality, a hard endpoint, is not very likely to be influenced, it could have affected the functional score on the mRS, which is a less objective endpoint. Meanwhile, a subjective bias might have occurred by unblinded diagnosis of infection rate, because infections could be diagnosed more easily in the control group, or less easily in the group treated with preventive antibiotic.

Among the six studies included, only two reported the figures of late infections, which limited further analysis on the effect of preventive antibiotic on late infections. Due to the fact that late infections are more often caused by nosocomial multidrug-resistant bacteria, they are probably more difficult to cure. If late infections and overall infections, not just early ones, can be further analyzed, this may help address this issue. This is a shortcoming of the current meta-analysis. Future studies are warranted to further focus on this specific issue.

Additionally, bias might be introduced when participants are withdrawn after randomization. Excluding participants because of an inability to complete the course due to side effects from antibiotics might produce bias in favor of the group treated with preventive antibiotic.

## Conclusions

The present meta-analysis of 6 randomized controlled trials including 4125 patients shows that although preventive antibiotic treatment did reduce the occurrence of early overall infections, early pneumonia and early UTI in adults with acute stroke, this advantage was not eventually translated to a shortened hospital stay, favorable outcome and reduction in mortality. Preventive antibiotics are unlikely to have an effect on favorable outcome or mortality in patients with acute stroke. Future studies are warranted to further focus on the late infections and overall incidence of infections, and identify any subgroup of stroke patients who might benefit from preventive antibiotic treatment.

## Supporting information

S1 File Definition of infection in each study. (PDF)

S2 File Forest plot of each outcome. (PDF)

S3 File Search strategy. (PDF)

S4 File PRISMA checklist. (PDF)
