# Supplementary material for: Should preventive antibiotics be used in patients with acute stroke? A systematic review and meta-analysis of randomized controlled trials
Source: PLoS One. 2017 Oct 19;12(10):e0186607. doi: 10.1371/journal.pone.0186607 (PMC5648227; doi:10.1371/journal.pone.0186607)
Supplement: S1 File — (PDF) [file pone.0186607.s001.pdf]

Table I Definition used for infection in each study

Study	Definition of infection
Chamorro 2005	temperature 37.5℃ in 2 determinations or 37.8℃ in a single determination in patients with suggestive symptoms (ie, cough, dyspnea, pleuritic pain, urinary tract symptoms),white blood cell count 11 000/mL or 4000/mL, pulmonary infiltrate on chest x-rays, or cultures positive for a pathogen.
Harms 2008	A) abnormal respiratory examination, pulmonary infiltrates in chest x-rays; B) productive cough with purulent sputum, microbiological cultures from lower respiratory tract or blood cultures, leukocytosis, and elevation of C-reactive protein. Diagnosis of urinary tract infection was based on two of the following criteria: fever (＞38℃), urine sample positive for nitrite, leucocyturia, and significant bacteriuria.
Kalra 2015	(1)patient`s temperature of at least 37. 5℃ or higher on two consecutive measurements or one measurement of 38. 0℃ or higher and (2) a respiratory rate of 20 breaths per min or more, or cough and breathlessness, or purulent sputum, and (3) a white blood cell count that is higher than 11. 0×10^{9}/L, or chest infiltrates on radiograph, or positive sputum culture or microbiology, or positive blood culture.
Kohler 2013	Not mentioned
Schwarz 2008	Pneumonia:<br>Evidence of a new infiltrate on the chest x-ray compatible with the diagnosis of infection plus at least one of the following findings:<br>Fever (temperature 38.0℃)<br>Leukocytosis 12000/L or leukopenia 3000/L<br>Purulent tracheal secretions<br>Tracheobronchitis<br>Purulent tracheals secretions or sputum plus at least one of the following findings:<br>Fever (temperature 38.0℃)<br>Leukocytosis 12000/L or leukopenia 3000/L<br>Urinary tract infection<br>Evidence of 25 leukocytes/L in the urine if not explained by other findings (eg, blood contamination); each urinary tract infection in this patient group is considered significant Bacteremia Evidence of bacteria in blood cultures<br>Sepsis<br>Clinical evidence of an infection with at least two of the following findings:<br>Temperature 38℃ or 35℃<br>Tachycardia 90/min<br>Tachypnea 20/min<br>Leukocytosis 12 000/L or leukopenia 3000/L

Westendorp 2014	Infection of unclear origin or other infections<br>Clinical evidence of an infection of unknown origin or any other systemic infection<br>according to modified Centers for Disease Control and Prevention criteria (2008).
