# Supplementary material for: Should preventive antibiotics be used in patients with acute stroke? A systematic review and meta-analysis of randomized controlled trials
Source: PLoS One. 2017 Oct 19;12(10):e0186607. doi: 10.1371/journal.pone.0186607 (PMC5648227; doi:10.1371/journal.pone.0186607)

Forest plot of each outcome

Fig. I Early infection

A

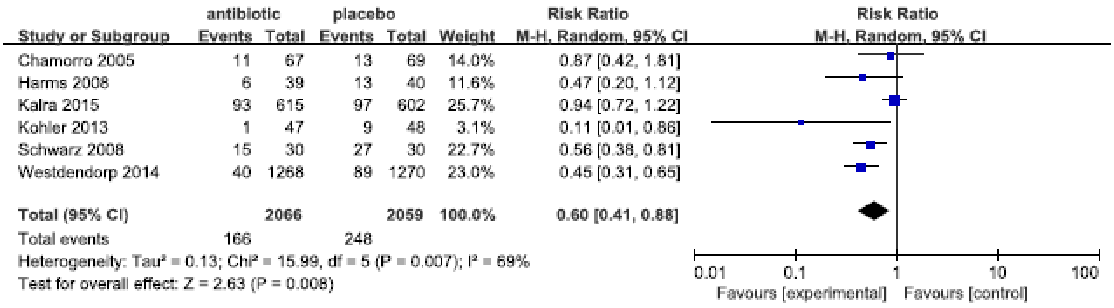

B

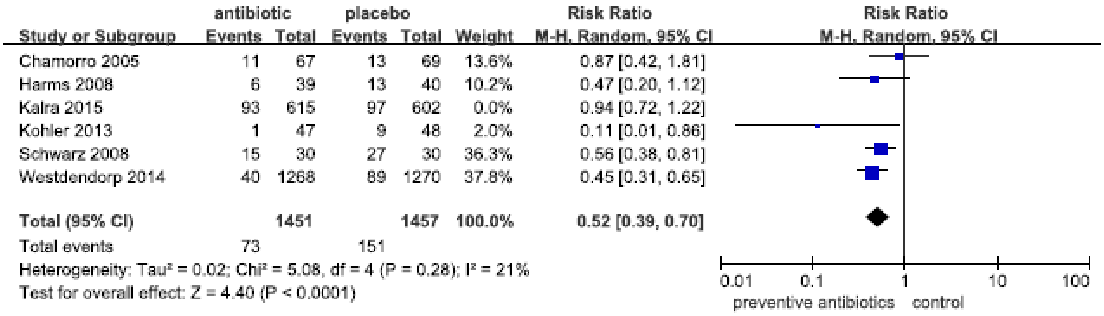

A: Forest plot of early infection. B: Sensitivity analysis for early infection.

Fig. II Early pneumonia

A

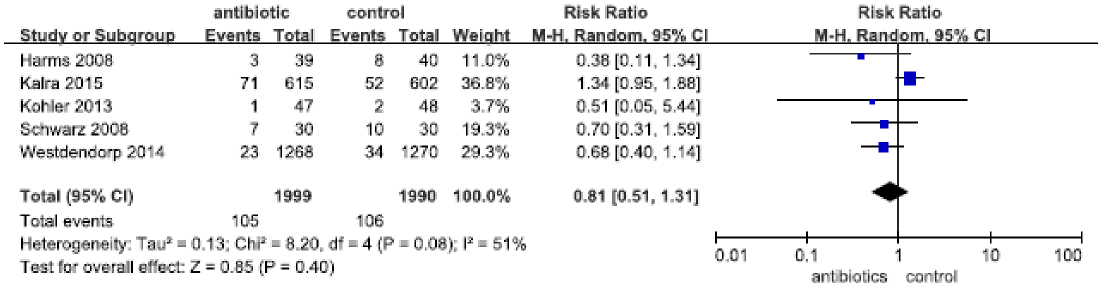

B

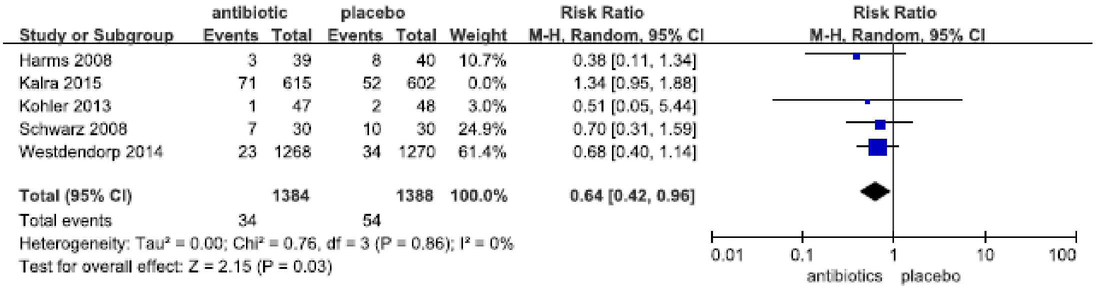

A: Forest plot of early pneumonia. B: Sensitivity analysis for early pneumonia.

Fig. III Early urinary tract infection

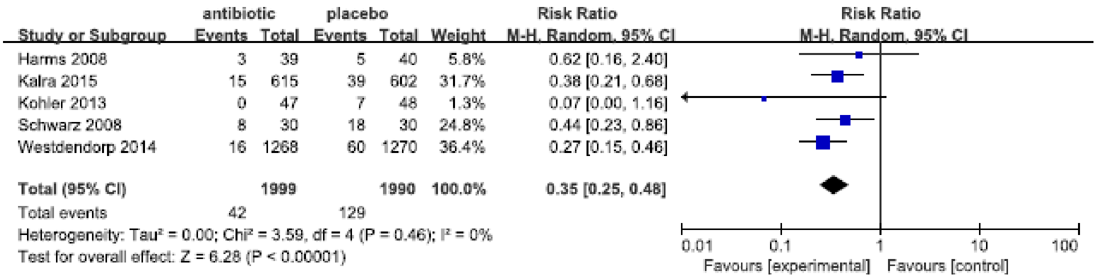

Fig. IV Overall mortality

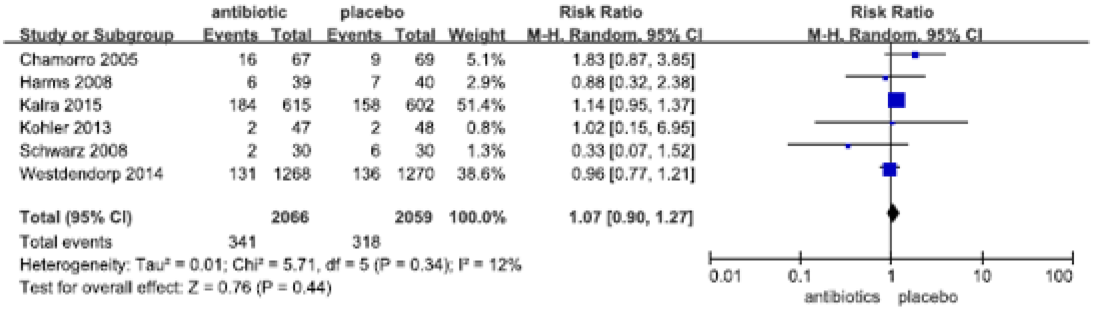

Fig. V Early mortality

Study or Subgroup	antibiotic Events	antibiotic Total	placebo Events	placebo Total	Weight	Risk Ratio M-H, Random, 95% CI
Harms 2008	3	39	4	40	2.8%	0.77 [0.18, 3.22]
Kalra 2015	62	615	56	602	48.9%	1.08 [0.77, 1.53]
Kohler 2013	1	47	1	48	0.8%	1.02 [0.07, 15.86]
Schwarz 2008	1	30	3	30	1.2%	0.33 [0.04, 3.03]
Westdendorp 2014	57	1268	61	1270	46.4%	0.94 [0.66, 1.33]
**Total (95% CI)**		**1999**		**1990**	**100.0%**	**0.99 [0.78, 1.26]**
Total events	124		125			

Heterogeneity: $Tau^2 = 0.00$; $Chi^2 = 1.42$, df = 4 (P = 0.84); $I^2 = 0\%$
Test for overall effect: Z = 0.10 (P = 0.92)

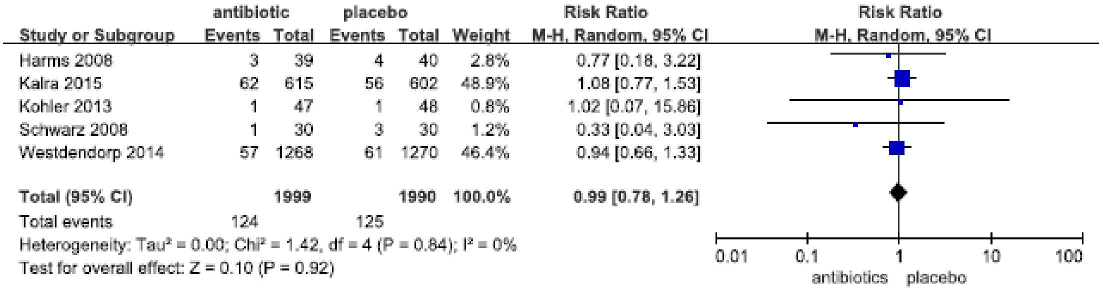

Fig. VI Late mortality

Study or Subgroup	antibiotic Events	antibiotic Total	placebo Events	placebo Total	Weight	Risk Ratio M-H, Random, 95% CI
Chamorro 2005	16	67	9	69	5.9%	1.83 [0.87, 3.85]
Harms 2008	3	39	3	40	1.4%	1.03 [0.22, 4.78]
Kalra 2015	122	615	102	602	57.9%	1.17 [0.92, 1.49]
Kohler 2013	1	47	1	48	0.4%	1.02 [0.07, 15.86]
Schwarz 2008	1	30	3	30	0.7%	0.33 [0.04, 3.03]
Westdendorp 2014	74	1268	75	1270	33.7%	0.99 [0.72, 1.35]
**Total (95% CI)**		**2066**		**2059**	**100.0%**	**1.12 [0.94, 1.35]**
Total events	217		193			

Heterogeneity: $Tau^2 = 0.00$; $Chi^2 = 3.61$, df = 5 (P = 0.61); $I^2 = 0\%$
Test for overall effect: Z = 1.26 (P = 0.21)

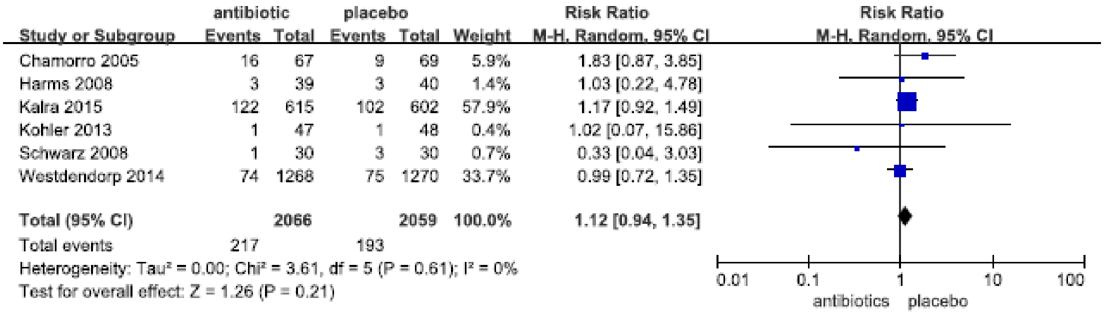

Fig. VII Favorable outcome

Study or Subgroup	antibiotic Events	antibiotic Total	placebo Events	placebo Total	Weight	Risk Ratio M-H, Random, 95% CI
Kalra 2015	109	615	121	602	11.0%	0.88 [0.70, 1.11]
Kohler 2013	29	47	33	48	7.0%	0.90 [0.67, 1.21]
Schwarz 2008	0	30	0	30		Not estimable
Westdendorp 2014	781	1268	763	1270	82.0%	1.03 [0.96, 1.09]
**Total (95% CI)**		**1960**		**1950**	**100.0%**	**1.00 [0.92, 1.08]**
Total events	919		917			

Heterogeneity: $Tau^2 = 0.00$; $Chi^2 = 2.21$, df = 2 (P = 0.33); $I^2 = 10\%$
Test for overall effect: Z = 0.02 (P = 0.98)

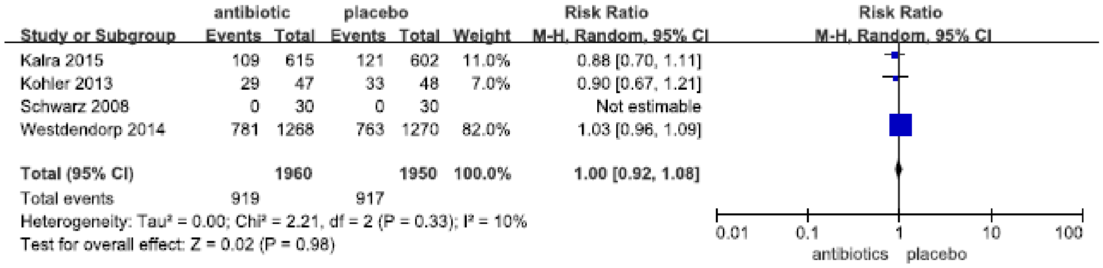

Fig. VIII Outcome in ischemic stroke

Study or Subgroup	antibiotic Events	antibiotic Total	placebo Events	placebo Total	Weight	Risk Ratio M-H, Random, 95% CI
Kohler 2013	27	39	28	38	5.3%	0.94 [0.71, 1.25]
Schwarz 2008	0	30	0	30		Not estimable
Westdendorp 2014	663	1058	646	1067	94.7%	1.04 [0.97, 1.11]
**Total (95% CI)**		**1127**		**1135**	**100.0%**	**1.03 [0.96, 1.10]**
Total events	690		674			

Heterogeneity: $Tau^2 = 0.00$; $Chi^2 = 0.43$, df = 1 (P = 0.51); $I^2 = 0\%$
Test for overall effect: Z = 0.88 (P = 0.38)

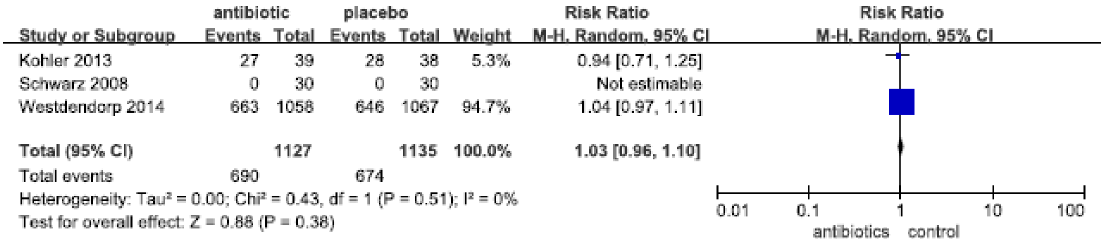

Fig. IX Outcome in hemorrhagic stroke

Study or Subgroup	antibiotic Events	antibiotic Total	placebo Events	placebo Total	Weight	Risk Ratio M-H, Random, 95% CI
Kohler 2013	2	5	2	6	2.8%	1.20 [0.25, 5.71]
Westdendorp 2014	61	143	59	126	97.2%	0.91 [0.70, 1.19]
**Total (95% CI)**		**148**		**132**	**100.0%**	**0.92 [0.71, 1.19]**
Total events	63		61			

Heterogeneity: Tau² = 0.00; Chi² = 0.12, df = 1 (P = 0.73); I² = 0%
Test for overall effect: Z = 0.64 (P = 0.52)

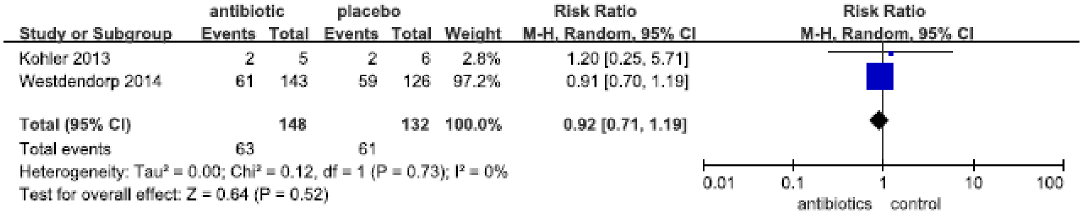

Supplement: S2 File — (PDF) [file pone.0186607.s002.pdf]
