# Supplementary material for: Should preventive antibiotics be used in patients with acute stroke? A systematic review and meta-analysis of randomized controlled trials
Source: PLoS One. 2017 Oct 19;12(10):e0186607. doi: 10.1371/journal.pone.0186607 (PMC5648227; doi:10.1371/journal.pone.0186607)
Supplement: S3 File — (PDF) [file pone.0186607.s003.pdf]

Table II. Search strategy

Database	Search strategy
Pubmed	Search ((″Stroke″[Mesh]) OR ″Cerebrovascular Disorders″[Mesh]) OR ″Basal Ganglia Cerebrovascular Disease″[Mesh]) OR ″Brain Ischemia″[Mesh]) OR ″Carotid Artery Diseases″[Mesh]) OR ″Cerebrovascular Trauma″[Mesh]) OR ″Intracranial Arterial Diseases″[Mesh]) OR ″Intracranial Arteriovenous Malformations″[Mesh]) OR (″Intracranial Embolism and Thrombosis″[Mesh])) OR ″Intracranial Hemorrhages″[Mesh]) OR ″Intracranial Hemorrhage, Hypertensive″[Mesh]) OR ″Brain Infarction″[Mesh]) OR ″Vasospasm, Intracranial″[Mesh]) OR ″Vertebral Artery Dissection″[Mesh]) OR ″Carotid Artery, Internal, Dissection″[Mesh]))))) OR ((((stroke*[Title/Abstract]) OR poststroke*[Title/Abstract]) OR cva*[Title/Abstract]) OR cerebrovascular*[Title/Abstract]) OR cerebral vascular[Title/Abstract])) OR ((((((cerebral[Title/Abstract]) OR cerebellar[Title/Abstract]) OR brain*[Title/Abstract]) OR vertebrobasilar[Title/Abstract])) AND ((((((infarct*[Title/Abstract]) OR ischaemi*[Title/Abstract]) OR ischemi*[Title/Abstract]) OR thrombo*[Title/Abstract]) OR apoplexy[Title/Abstract]) OR emboli*[Title/Abstract]))))) OR (((((((cerebral[Title/Abstract]) OR intracerebral[Title/Abstract]) OR intracranial[Title/Abstract]) OR brain*[Title/Abstract]) OR cerebellar[Title/Abstract]) OR subarachnoid[Title/Abstract])) AND ((((((haemorrhage[Title/Abstract]) OR hemorrhage[Title/Abstract]) OR haematoma[Title/Abstract]) OR hematoma[Title/Abstract]) OR bleeding[Title/Abstract]) OR aneurysm*[Title/Abstract])))) AND ((″Antibiotic Prophylaxis″[Mesh]) OR ((((((″Anti-Bacterial Agents″[Mesh]) OR (((((((((antibiotic*[Title/Abstract]) OR anti-bacterial[Title/Abstract]) OR anti bacterial[Title/Abstract]) OR antibacterial[Title/Abstract]) OR bacteriocid*[Title/Abstract]) OR anti-

	mycobacterial[Title/Abstract]) OR anti mycobacterial[Title/Abstract]) OR antimycobacterial[Title/Abstract]) OR anti-infect*[Title/Abstract]) OR anti infect*[Title/Abstract])) OR ((((((((((((((((((((amoxicillin[Title/Abstract]) OR amphotericin b[Title/Abstract]) OR ampicillin[Title/Abstract]) OR calcimycin[Title/Abstract]) OR cephalosporin*[Title/Abstract]) OR cephalothin[Title/Abstract]) OR cephamycin*[Title/Abstract]) OR ceftriaxone[Title/Abstract]) OR chloramphenicol[Title/Abstract]) OR dactinomycin[Title/Abstract]) OR doxycycline[Title/Abstract]) OR erythromycin[Title/Abstract]) OR fluoroquinolone*[Title/Abstract]) OR gentamicin*[Title/Abstract]) OR kanamycin[Title/Abstract]) OR minocycline[Title/Abstract]) OR neomycin[Title/Abstract]) OR oxytetracycline[Title/Abstract]) OR penicillin[Title/Abstract]) OR streptomycin[Title/Abstract]) OR tetracycline[Title/Abstract]) OR vancomycin[Title/Abstract]))) AND (((((((″Infection″[Mesh]) OR ″Bacterial Infections″[Mesh]) OR ″Infection Control″[Mesh]) OR ″Fever″[Mesh]) OR ″Inflammation″[Mesh])) OR (((((((((infection*[Title/Abstract]) OR sepsis[Title/Abstract]) OR septicaemia[Title/Abstract]) OR septicemia[Title/Abstract]) OR pneumonia[Title/Abstract]) OR bacteremia[Title/Abstract]) OR bacteraemia[Title/Abstract]) OR inflammation[Title/Abstract]) OR fever[Title/Abstract]) OR blood poisoning[Title/Abstract]))) AND (((((((prophyla*[Title/Abstract]) OR prevent*[Title/Abstract]) OR premedicat*[Title/Abstract]) OR incidence[Title/Abstract]) OR occurrence[Title/Abstract])) OR (″prevention and control″ [Subheading]))))))))) AND

	((((((((((((((″Randomized Controlled Trials as Topic″[Mesh]) OR ″Random Allocation″[Mesh]) OR ″Double-Blind Method″[Mesh]) OR ″Single-Blind Method″[Mesh]) OR (″Clinical Trial, Phase I″ [Publication Type] OR ″Clinical Trial, Phase II″ [Publication Type] OR ″Clinical Trial, Phase III″ [Publication Type] OR ″Clinical Trial, Phase IV″ [Publication Type])) OR ″Controlled Clinical Trial″ [Publication Type]) OR ″Randomized Controlled Trial″ [Publication Type]) OR ″Multicenter Study″ [Publication Type]) OR ″Clinical Trial″ [Publication Type]) OR ″Clinical Trials as Topic″[Mesh])) OR (((((((clinical[Title/Abstract]) AND trial*[Title/Abstract])) OR ((((((singl*[Title/Abstract]) OR doubl*[Title/Abstract]) OR treb*[Title/Abstract]) OR tripl*[Title/Abstract])) AND ((blind*[Title/Abstract]) OR mask*[Title/Abstract]))) OR ″Placebos″[Mesh]) OR placebo*[Title/Abstract]) OR random*[Title/Abstract]))))) Results Date 139 27 Feb 2017
Embase	Query Results Results: 1,373 Date : 27 Feb 2017 'cerebrovascular disease'/exp OR 'basal ganglion hemorrhage'/exp OR 'brain hematoma'/exp OR 'brain hemorrhage'/exp OR 'brain infarction'/exp OR 'brain ischemia'/exp OR 'carotid artery disease'/exp OR 'cerebral artery disease'/exp OR 'cerebrovascular accident'/exp OR 'cerebrovascular malformation'/exp OR 'intracranial aneurysm'/exp OR 'occlusive cerebrovascular disease'/exp OR 'stroke patient'/exp OR 'stroke unit'/exp OR stroke*:ab,ti OR poststroke*:ab,ti OR cva*:ab,ti OR cerebrovascular*:ab,ti OR (cerebral AND vascular:ab,ti) OR (cerebral:ab,ti OR cerebellar:ab,ti OR brain*:ab,ti OR vertebrobasilar:ab,ti AND (infarct*:ab,ti OR ischaemi*:ab,ti OR ischemi*:ab,ti OR thrombo*:ab,ti OR apoplexy:ab,ti OR emboli*:ab,ti)) OR (cerebral:ab,ti OR intracerebral:ab,ti OR intracranial:ab,ti OR brain*:ab,ti OR cerebellar:ab,ti OR

	subarachnoid:ab,ti AND (haemorrhage:ab,ti OR hemorrhage:ab,ti OR haematoma:ab,ti OR hematoma:ab,ti OR bleeding:ab,ti OR aneurysm*:ab,ti)) AND ('antibiotic prophylaxis'/exp OR ('antibiotic agent'/exp OR antibiotic*:ab,ti OR 'anti bacterial':ab,ti OR (anti AND bacterial:ab,ti) OR antibacterial:ab,ti OR bacteriocid*:ab,ti OR 'anti mycobacterial':ab,ti OR (anti AND mycobacterial:ab,ti) OR antimycobacterial:ab,ti OR 'anti infect':ab,ti OR 'anti infection':ab,ti OR 'anti infections':ab,ti OR 'anti infective':ab,ti OR 'anti infectives':ab,ti OR (anti AND infect*:ab,ti) OR amoxicillin:ab,ti OR 'amphotericin b':ab,ti OR ampicillin:ab,ti OR calcimycin:ab,ti OR cephalosporin*:ab,ti OR cephalothin:ab,ti OR cephamycin*:ab,ti OR ceftriaxone:ab,ti OR chloramphenicol:ab,ti OR dactinomycin:ab,ti OR doxycycline:ab,ti OR erythromycin:ab,ti OR fluoroquinolone*:ab,ti OR gentamicin*:ab,ti OR kanamycin*:ab,ti OR minocycline:ab,ti OR neomycin:ab,ti OR oxytetracycline:ab,ti OR penicillin:ab,ti OR streptomycin:ab,ti OR tetracycline:ab,ti OR vancomycin:ab,ti AND ('infection'/exp OR 'infection control'/exp OR 'infection risk'/exp OR 'fever'/exp OR 'inflammation'/exp OR infection*:ab,ti OR sepsis:ab,ti OR septicaemia:ab,ti OR septicemia:ab,ti OR pneumonia:ab,ti OR bacteremia:ab,ti OR bacteraemia:ab,ti OR inflammation:ab,ti OR fever:ab,ti OR (blood AND poisoning:ab,ti)) AND (prophyla*:ab,ti OR prevent*:ab,ti OR premedicat*:ab,ti OR incidence:ab,ti OR occurrence:ab,ti OR 'prophylaxis'/exp)) OR ('antibiotic agent'/exp OR antibiotic*:ab,ti OR 'anti bacterial':ab,ti OR (anti AND bacterial:ab,ti) OR antibacterial:ab,ti OR bacteriocid*:ab,ti OR 'anti mycobacterial':ab,ti OR (anti AND mycobacterial:ab,ti) OR antimycobacterial:ab,ti OR 'anti infect':ab,ti OR 'anti infection':ab,ti OR 'anti infections':ab,ti OR 'anti infective':ab,ti OR 'anti infectives':ab,ti OR (anti AND infect*:ab,ti) OR

	amoxicillin:ab,ti OR 'amphotericin b':ab,ti OR ampicillin:ab,ti OR calcimycin:ab,ti OR cephalosporin*:ab,ti OR cephalothin:ab,ti OR cephamycin*:ab,ti OR ceftriaxone:ab,ti OR chloramphenicol:ab,ti OR dactinomycin:ab,ti OR doxycycline:ab,ti OR erythromycin:ab,ti OR fluoroquinolone*:ab,ti OR gentamicin*:ab,ti OR kanamycin*:ab,ti OR minocycline:ab,ti OR neomycin:ab,ti OR oxytetracycline:ab,ti OR penicillin:ab,ti OR streptomycin:ab,ti OR tetracycline:ab,ti OR vancomycin:ab,ti AND ('infection prevention'/exp OR 'infection'/exp))) AND ('randomized controlled trial':it OR 'pragmatic clinical trial':it OR 'randomized controlled trial (topic)'/exp OR 'randomized controlled trial'/exp OR 'randomization'/exp OR 'double blind procedure'/exp OR 'single blind procedure'/exp OR 'placebo'/exp OR random*:ab,ti OR sham:ab,ti OR placebo*:ab,ti OR (singl*:ab,ti OR doubl*:ab,ti AND (blind*:ab,ti OR dumm*:ab,ti OR mask*:ab,ti)) OR (blind*:ab,ti OR dumm*:ab,ti OR mask*:ab,ti AND (tripl*:ab,ti OR trebl*:ab,ti)))
Cochrane library	Search Name: preventive antibiotics in patients with acute stroke<br>Last Saved: 27/02/2017 17:24:30.024 Results: 163<br>Description:<br><br>ID Search<br>#1 MeSH descriptor: [Stroke] explode all trees<br>#2 MeSH descriptor: [Cerebrovascular Disorders] explode all trees<br>#3 MeSH descriptor: [Basal Ganglia Cerebrovascular Disease] explode all trees<br>#4 MeSH descriptor: [Brain Ischemia] explode all trees<br>#5 MeSH descriptor: [Carotid Artery Diseases] explode all trees<br>#6 MeSH descriptor: [Cerebrovascular Trauma] explode all trees<br>#7 MeSH descriptor: [Intracranial Arterial Diseases] explode all trees<br>#8 MeSH descriptor: [Intracranial Arteriovenous Malformations] explode all trees<br>#9 MeSH descriptor: [Intracranial Embolism and Thrombosis] explode all trees<br>#10 MeSH descriptor: [Intracranial Hemorrhages] explode all trees<br>#11 MeSH descriptor: [Intracranial Hemorrhage, Hypertensive] explode all trees<br>#12 MeSH descriptor: [Brain Infarction] explode all trees

	#13 MeSH descriptor: [Vasospasm, Intracranial] explode all trees<br>#14 MeSH descriptor: [Vertebral Artery Dissection] explode all trees<br>#15 MeSH descriptor: [Carotid Artery, Internal, Dissection] explode all trees<br>#16 #1 or #2 or #3 or #4 or #5 or #6 or #7 or #8 or #9 or #10 or #11 or #12 or #13 or #14 or #15<br>#17 stroke*:ti,ab,kw (Word variations have been searched)<br>#18 poststroke*:ti,ab,kw (Word variations have been searched)<br>#19 cva*:ti,ab,kw (Word variations have been searched)<br>#20 cerebrovascular*:ti,ab,kw (Word variations have been searched)<br>#21 cerebral vascular:ti,ab,kw (Word variations have been searched)<br>#22 #17 or #18 or #19 or #20 or #21<br>#23 cerebral:ti,ab,kw (Word variations have been searched)<br>#24 cerebellar:ti,ab,kw (Word variations have been searched)<br>#25 brain*:ti,ab,kw (Word variations have been searched)<br>#26 vertebrobasilar:ti,ab,kw (Word variations have been searched)<br>#27 #23 or #24 or #25 or #26<br>#28 infarct*:ti,ab,kw (Word variations have been searched)<br>#29 ischaemi*:ti,ab,kw (Word variations have been searched)<br>#30 ischemi*:ti,ab,kw (Word variations have been searched)<br>#31 thrombosis:ti,ab,kw (Word variations have been searched)<br>#32 apoplexy:ti,ab,kw (Word variations have been searched)<br>#33 emboli*:ti,ab,kw (Word variations have been searched)<br>#34 #28 or #29 or #30 or #31 or #32 or #33<br>#35 #27 and #34<br>#36 cerebral:ti,ab,kw (Word variations have been searched)<br>#37 intracerebral:ti,ab,kw (Word variations have been searched)<br>#38 intracranial:ti,ab,kw (Word variations have been searched)<br>#39 brain*:ti,ab,kw (Word variations have been searched)<br>#40 cerebellar:ti,ab,kw (Word variations have been searched)<br>#41 subarachnoid:ti,ab,kw (Word variations have been searched)<br>#42 #36 or #37 or #38 or #39 or #40 or #41<br>#43 haemorrhage:ti,ab,kw (Word variations have been searched)<br>#44 hemorrhage:ti,ab,kw (Word variations have been searched)<br>#45 haematoma:ti,ab,kw (Word variations have been searched)<br>#46 hematoma:ti,ab,kw (Word variations have been searched)<br>#47 bleeding:ti,ab,kw (Word variations have been searched)<br>#48 aneurysm*:ti,ab,kw (Word variations have been searched)<br>#49 #43 or #44 or #45 or #46 or #47 or #48<br>#50 #42 and #49<br>#51 #16 or #22 or #35 or #50<br>#52 MeSH descriptor: [Antibiotic Prophylaxis] explode all trees<br>#53 MeSH descriptor: [Anti-Bacterial Agents] explode all trees<br>#54 antibiotic*:ti,ab,kw (Word variations have been searched)

	#55 anti-bacterial:ti,ab,kw (Word variations have been searched)<br>#56 anti bacterial:ti,ab,kw (Word variations have been searched)<br>#57 antibacterial:ti,ab,kw (Word variations have been searched)<br>#58 bacteriocid*:ti,ab,kw (Word variations have been searched)<br>#59 anti-mycobacterial:ti,ab,kw (Word variations have been searched)<br>#60 anti mycobacterial:ti,ab,kw (Word variations have been searched)<br>#61 antimycobacterial:ti,ab,kw (Word variations have been searched)<br>#62 anti-infect*:ti,ab,kw (Word variations have been searched)<br>#63 anti infect*:ti,ab,kw (Word variations have been searched)<br>#64 #54 or #55 or #56 or #57 or #58 or #59 or #60 or #61 or #62 or #63<br>#65 amoxicillin:ti,ab,kw (Word variations have been searched)<br>#66 amphotericin b:ti,ab,kw (Word variations have been searched)<br>#67 ampicillin:ti,ab,kw (Word variations have been searched)<br>#68 calcimycin:ti,ab,kw (Word variations have been searched)<br>#69 cephalosporin*:ti,ab,kw (Word variations have been searched)<br>#70 cephalothin:ti,ab,kw (Word variations have been searched)<br>#71 cephamycin*:ti,ab,kw (Word variations have been searched)<br>#72 ceftriaxone:ti,ab,kw (Word variations have been searched)<br>#73 chloramphenicol:ti,ab,kw (Word variations have been searched)<br>#74 dactinomycin:ti,ab,kw (Word variations have been searched)<br>#75 doxycycline:ti,ab,kw (Word variations have been searched)<br>#76 erythromycin:ti,ab,kw (Word variations have been searched)<br>#77 fluoroquinolone*:ti,ab,kw (Word variations have been searched)<br>#78 gentamicin*:ti,ab,kw (Word variations have been searched)<br>#79 kanamycin:ti,ab,kw (Word variations have been searched)<br>#80 minocycline:ti,ab,kw (Word variations have been searched)<br>#81 neomycin:ti,ab,kw (Word variations have been searched)<br>#82 oxytetracycline:ti,ab,kw (Word variations have been searched)<br>#83 penicillin:ti,ab,kw (Word variations have been searched)<br>#84 streptomycin:ti,ab,kw (Word variations have been searched)<br>#85 tetracycline:ti,ab,kw (Word variations have been searched)<br>#86 vancomycin:ti,ab,kw (Word variations have been searched)<br>#87 #65 or #66 or #67 or #68 or #69 or #70 or #71 or #72 or #73 or #74 or #75 or #76 or #77 or #78 or #79 or #80 or #81 or #82 or #83 or #84 or #85 or #86<br>#88 #53 or #64 or #87<br>#89 MeSH descriptor: [Infection] explode all trees<br>#90 MeSH descriptor: [Bacterial Infections] explode all trees<br>#91 MeSH descriptor: [Infection Control] explode all trees<br>#92 MeSH descriptor: [Fever] explode all trees<br>#93 MeSH descriptor: [Inflammation] explode all trees<br>#94 #89 or #90 or #91 or #92 or #93<br>#95 infection*:ti,ab,kw (Word variations have been searched)<br>#96 sepsis:ti,ab,kw (Word variations have been searched)

	#97 septicaemia:ti,ab,kw (Word variations have been searched)<br>#98 septicemia:ti,ab,kw (Word variations have been searched)<br>#99 pneumonia:ti,ab,kw (Word variations have been searched)<br>#100 bacteremia:ti,ab,kw (Word variations have been searched)<br>#101 bacteraemia:ti,ab,kw (Word variations have been searched)<br>#102 inflammation:ti,ab,kw (Word variations have been searched)<br>#103 fever:ti,ab,kw (Word variations have been searched)<br>#104 blood poisoning:ti,ab,kw (Word variations have been searched)<br>#105 #95 or #96 or #97 or #98 or #99 or #100 or #101 or #102 or #103 or #104<br>#106 #94 or #105<br>#107 prophyla*:ti,ab,kw (Word variations have been searched)<br>#108 prevent*:ti,ab,kw (Word variations have been searched)<br>#109 premedicat*:ti,ab,kw (Word variations have been searched)<br>#110 incidence:ti,ab,kw (Word variations have been searched)<br>#111 occurrence:ti,ab,kw (Word variations have been searched)<br>#112 #107 or #108 or #109 or #110 or #111<br>#113 #88 and #106 and #112<br>#114 #52 or #113<br>#115 #51 and #114
